# Design of Intelligent Detection Platform for Wine Grape Pests and Diseases in Ningxia

**DOI:** 10.3390/plants12010106

**Published:** 2022-12-26

**Authors:** Yutan Wang, Chi Wei, Haowei Sun, Aili Qu

**Affiliations:** Department of Mechanical Engineering, Ningxia University, Yinchuan 750000, China

**Keywords:** wine grape pest, deep learning, target detection, YOLOX_s, VNC remote monitoring

## Abstract

In order to reduce the impact of pests and diseases on the yield and quality of Ningxia wine grapes and to improve the efficiency and intelligence of detection, this paper designs an intelligent detection platform for pests and diseases. The optimal underlying network is selected by comparing the recognition accuracy of both MobileNet V2 and YOLOX_s networks trained on the Public Dataset. Based on this network, the effect of adding attention mechanism and replacing loss function on recognition effect is investigated by permutation in the Custom Dataset, resulting in the improved network YOLOX_s + CBAM. The improved network was trained on the Overall Dataset, and finally a recognition model capable of identifying nine types of pests was obtained, with a recognition accuracy of 93.35% in the validation set, an improvement of 1.35% over the original network. The recognition model is deployed on the Web side and Raspberry Pi to achieve independent detection functions; the channel between the two platforms is built through Ngrok, and remote interconnection is achieved through VNC desktop. Users can choose to upload local images on the Web side for detection, handheld Raspberry Pi for field detection, or Raspberry Pi and Web interconnection for remote detection.

## 1. Introduction

In terms of target detection algorithms, a lot of research has been conducted by many scholars. Jaime et al. [1] proposed a wireless sensor based on IEEE802.11a/b/g/n transmission standard for image acquisition and real-time monitoring of vineyard site conditions. Since its image processing system detects diseases by setting the threshold of Hue Saturation Value (HSV), it failed to identify those diseases with HSV values close to healthy leaves, such as grape downy mildew, sunburn, etc. Bharate et al. [2] used K-nearest Neighbor (KNN) to classify the given grape leaves as healthy and non-healthy with an accuracy of 90%. Ntihemuka et al. [3] combined wireless sensor network (WSN) with KNN classification algorithm and Ran learning algorithm in machine learning to build a farm environment monitoring platform that can monitor eight environmental parameters related to pests and diseases in real time. However, these algorithms, KNN and RAN, are unable to extract features effectively and have weaker generalization ability than convolutional neural networks such as CNN and DCNN.

Militante et al. [4] used an improved CNN model to detect and identify diseases in six crops, including grapes, apples, corn, and others, achieving 96.5% accuracy. Rajinder et al. [5] used a deep convolutional neural network (DCNN) model to identify three grape diseases the model with an accuracy of 99.34%. Xiaoyue et al. [6] introduced the ResNet-v2 module and SE module on the basis of Faster R-CNN network and proposed the DR-IACNN model for detecting four common grape leaf diseases. The model has stronger feature extraction capability, and it achieves 81.1% detection accuracy on the grape leaf disease dataset. Singh et al. [7] proposed a method to detect black measles disease from grape leaf image samples using Support Vector Machines (SVM) with an accuracy of 97.2%, but a single type of disease was detected. Juanhua et al. [8] used the Prewitt Operator to extract the complete edge of the lesion region, extracted five effective feature parameters such as perimeter, area, and roundness, and proposed an automatic grape leaf disease detection model based on back propagation neural network (BPNN), which can effectively detect and identify five grape leaf diseases with an average correct rate of 91%. Syed et al. [9] compared the performance of five different deep learning models for rice paddy disease detection, namely Vgg16 [10], Vgg19 [11], ResNet50 [12], ResNet50V2, and ResNet101V2. Among them, the ResNet101V2 model performed the best with an accuracy of 86.799%. Although the CNN, Faster R-CNN, and ResNet networks have high recognition accuracy, the large number of network parameters and long training time make them unsuitable for deployment on mobile platforms.

Considering the large number of pest species detected in this experiment and the long training time, a more lightweight network should be selected to ensure the appropriate accuracy and improve the training speed of the model. Rui Yang et al. [13] combined RGB images (RGBI), multispectral images (MSI), and thermal infrared images (Tiri) of leaf pests and diseases into a multi-source data connection (MDC) model with 31 channels by acquiring, and proposed a multi-source data fusion (MDF) decision method based on ShuffleNetV2 [14] lightweight network to improve the detection performance of five grape leaf diseases with an overall accuracy of 96.05%. Although the MDF method has high accuracy with low number of network parameters and high parallel computing capability, it increases the difficulty of sample production and prolongs the model training time due to the need to extract three dimensions of information on the target simultaneously. Andler et al. [15] proposed the MobileNet V2 model, which enhances the gradient propagation by introducing the inverse residual structure and significantly reduces the memory usage during inference, with 9 times fewer parameters than SSD300 and SSD512, which is very suitable for small mobile devices such as Raspberry Pi and cell phones, and meets the design requirements of this experiment.

The YOLO series is improved from R-CNN, and the detection speed is greatly improved by regression processing of target border and classification probability, which is suitable for real-time detection tasks. Jun et al. [16] used image pyramid feature fusion to achieve the extraction of multi-scale features, and the detection accuracy of the improved YOLOv3 model was 92.39%, which was 1.72% and 4.08% better than the Faster R-CNN and the original network, respectively. Yan Zhang et al. [17] proposed an improved YOLOv5 to detect closed cotton boll by combining density network and attention mechanism, and the model reduced 37.3 Mb and 91.8 Mb compared with SSD-VGG16 and FasterRCN-Inceptionv2, respectively, with 17.78% and 17.33% improvement in accuracy. YOLOX achieved 50.0% AP on COCO (1.8% higher AP than YOLOv5) in YOLOv4 and v5 with similar number of parameters, and YOLOX was able to achieve 68.9 FPS on a single Tesla V100, which has extremely high real-time detection performance [18]. Therefore, YOLOX with better performance was chosen as the base network for this experiment.

For the application of detection networks, Ganesh et al. [19] designed Android mobile applications based on YOLO, R-CNN object detection and recognition to navigate indoors for people with visual impairment. Labiba et al. [20] designed an Android application to classify fruits and vegetables and implemented the classification function and target detection function by VGG-16 and YOLO, respectively, with an average accuracy of 84%. Michael et al. [21] built an annotated dataset of Arabidopsis plants, trained it by Tiny-YOLOv3 network, and implanted the training model into an Android phone, which could accurately localize and count leaves in real time with inference time under 0.01 s. Ing-Chau et al. [22] developed a vehicle blind spot warning (PBSW) system based on YOLOv4. The detection system consisting of RaspberryPi, cell phone, and cloud server feeds the safety distance information between vehicles to the cell phone application with 92.82% correct estimated distance. However, the system process is too cumbersome, and it is possible to skip the mobile application and cloud processing ring to embed YOLO directly into the Raspberry Pi system. Gokulr et al. [23] use TensorFlow and Keras to build a CNN model to detect the distance between face masks, process the distance information through Firebase, and push the violation information to the Android-based StaySafe application.

This paper intends to select MobileNet V2 network and YOLOX_s, the smallest number of parameters in YOLOX series [18], as the base network and through further comparison, to explore the best detection model. It combines hardware and network communication technology to realize the application of neural network learning in the field of grape pest detection, providing a reference for the solution of pest detection of other agricultural crops.

## 2. Materials and Methods

**Figure 1:**
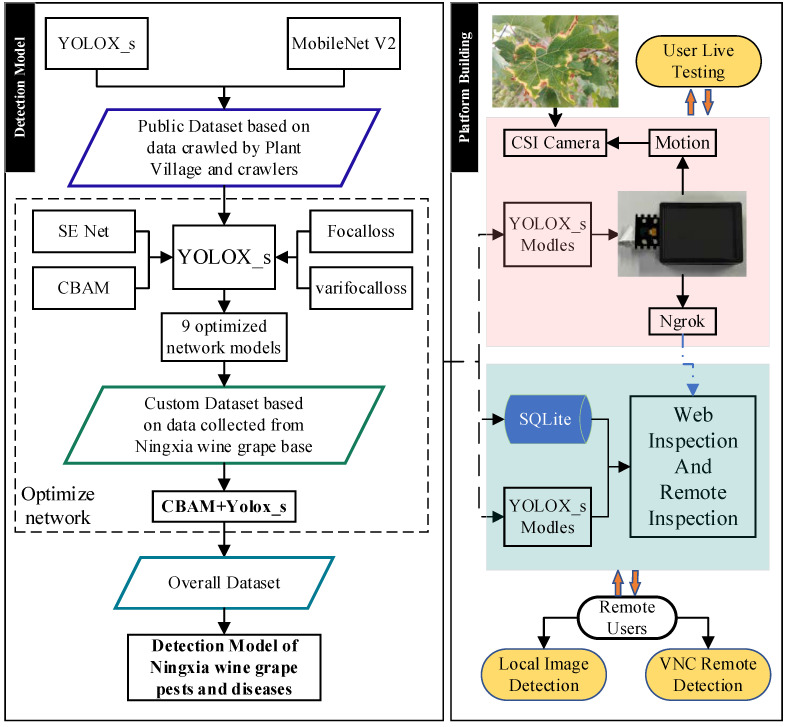
The technical route of network optimization and testing platform construction.

In this paper, three datasets are created according to the differences of experimental requirements and the applicable scenarios of each dataset are clarified. Firstly, a pre-test for cross-sectional comparison is designed to clarify the base detection network by comparing the detection accuracy of YOLOX_s and MobileNet V2 on different test items. Then, optimization for this base network occurs in various ways, while establishing an evaluation system to measure the effect of optimization. Based on the experimental results, the best performing improved network is selected and the final pest detection model is trained. Finally, the target detection module is deployed on the Web side and Raspberry Pi respectively to build the interconnection channel, and then the detection function of the Web platform, the on-site detection function, and the VNC remote real-time detection function are realized. The technical route is shown in Figure 1.

### 2.1. Construction of Dataset

**Figure 2:**
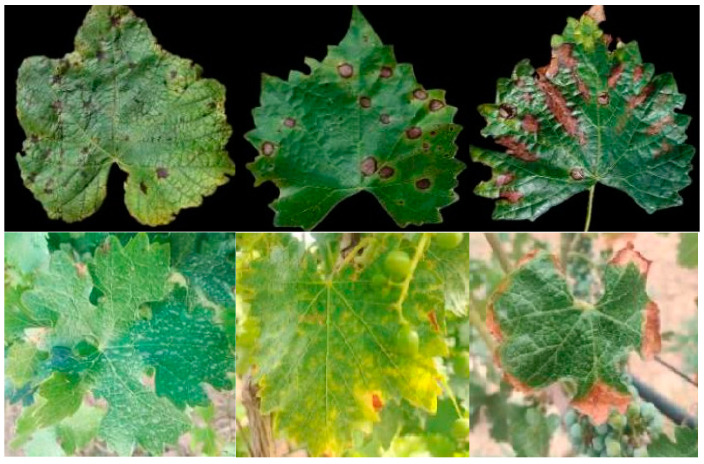
Grape pest and disease samples from overall dataset.

The datasets in this paper are divided into three categories: public dataset, custom dataset, and overall dataset. The samples of the dataset part are shown in Figure 2, and the characteristics of the dataset are shown in Table 1.

The public dataset was derived from the Python crawler and Plant Village dataset, which included 12 pests and diseases such as grape brown spot, black rot, and grapevine rash, with a total of 42,150 images. The custom dataset was collected from the grape growing base of Yuquanying Farm, Ningxia Province (106°1′35.75″ N, 38°15′46.14″ E) and the grape growing base of Luoshan Flying Camp, Ningxia Province (106°4′6.32″ N, 37°25′54.78″ E) from the beginning of May to the end of August 2020. The collection equipment was SKW-A0 and SONY CX610E. Nine pests and diseases were finally acquired, with a total of 21,850 images. The overall dataset was based on the self-defined dataset with samples of the same disease category in some public datasets, including a total of 32,760 pictures of 9 pests and diseases.

The public dataset is suitable for screening the underlying network. Having the largest number of disease species is suitable for testing the detection ability of the base network in a complex sample environment. It is also the least difficult to obtain and facilitates faster comparison of the base network before the collection of the custom dataset is completed. The custom dataset is used to filter the optimization network. It has a smaller sample size than the public dataset, but contains the characteristics of Ningxia pests and diseases, thus saving the time spent on training multiple optimization networks, while ensuring the relevance of the optimization model to Ningxia samples. The full dataset further expands the sample size while retaining the relevance of the custom dataset for Ningxia geographical samples, which is ideal for training the final identification model.

The operating system of this study is Windows 10, using PyTorch 1.10.0 framework, Anaconda3-5.1.0 (Python 3.7) environment, CUDA11.3 + CUDNN8.2.1, Intel Xeon-5118 CPU, Nvidia Titan RTX GPU, DRAM 64G. The initial resolution of the manually acquired images was 3000 × 4000 pixels, and the initial image volume was compressed to 640 × 640 and stored to improve the model training efficiency. The labeling of leaf pests and diseases was done using labelImg, and the ratio of training set to validation set was 8:2 for all three datasets.

### 2.2. Underlying Network Comparison Pre-Experiment

**Figure 3:**
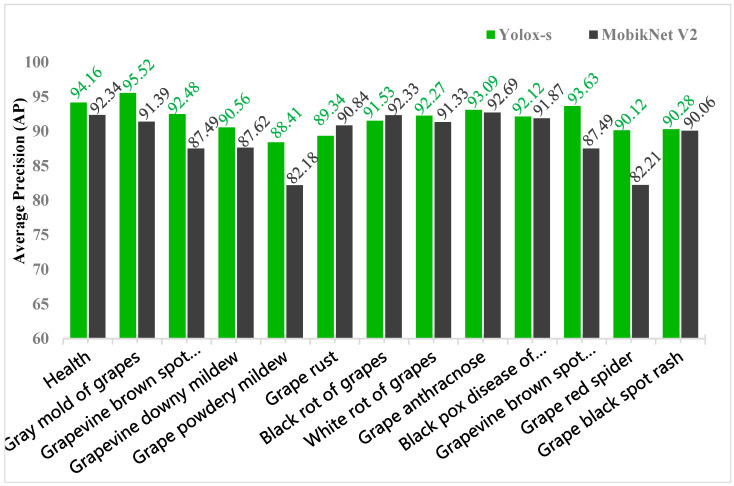
Average precision values of Yolox_s and MobileNet V2 detection for 12 pests and diseases in the public dataset.

The YOLOX_s network was trained with MobileNet V2 network under the Public Data Trainingset, where V2num_classes was set to 9, epoch value was set to 200, batch-size was set to 4, and input-size was defaulted [640, 640]. The detection results in the validation set of the public dataset are shown in Figure 3. The results showed that YOLOX_s had higher AP values than MobileNet V2 for all disease tests, except for grape rust and grape black rot, which had 1.5% and 0.8% lower AP values than MobileNet V2, respectively. YOLOX_s has better performance than MobileNet V2 with the same training set and settings, and it can maintain higher recognition accuracy in a wide variety of diseases. Therefore YOLOX_s is chosen as the underlying network.

### 2.3. Optimization of Yolox_s

**Figure 4:**
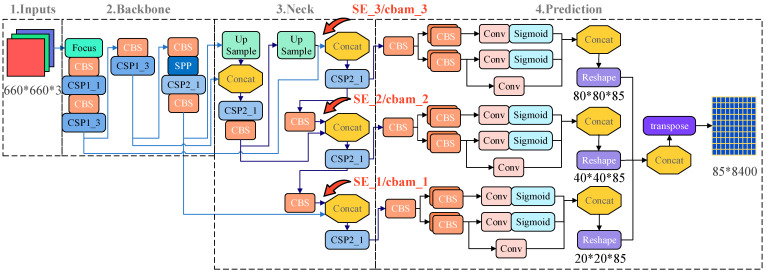
Schematic diagram of YOLOX_s network structure with SE Net and CBAM insertion.

YOLOX_s network is optimized by adding some tricks to Yolov5s. At the input side, YOLOX_s adds the ixup data enhancement effect on top of the Mosa data enhancement. In the Backbone and Neck parts, the activation functions both use SiLU functions. At the output side, the detection head is changed to Decoupled Head and uses anchor free, multi positives, SimOTA, etc. YOLOX_s uses 8×, 16× and 21× upsampling, i.e., the smallest pixels are 8 × 8, 16 × 16 and 32 × 32. These smallest pixels are used as feature points to form feature maps at different scales and are fused by means of FPN to form the final feature vector. The network structure is shown in Figure 4.

Since the actual area of the grape leaf pest is affected by the variation of the distance between the camera and the target, the area of the target cannot be used as the detection threshold of the metric system. In contrast, the target pixel size is more suitable as a metric for measuring the minimum detection threshold. Therefore, 8 × 8 pixels is used as the minimum value for the threshold of grape leaf pest detected by this network.

To meet the accuracy of actual grape pest detection, this paper intends to further improve the detection accuracy of the network by adding an appropriate attention mechanism or replacing the confidence function on top of the original YOLOX_s network.

#### 2.3.1. Attention Mechanism Optimization

**Figure 5:**
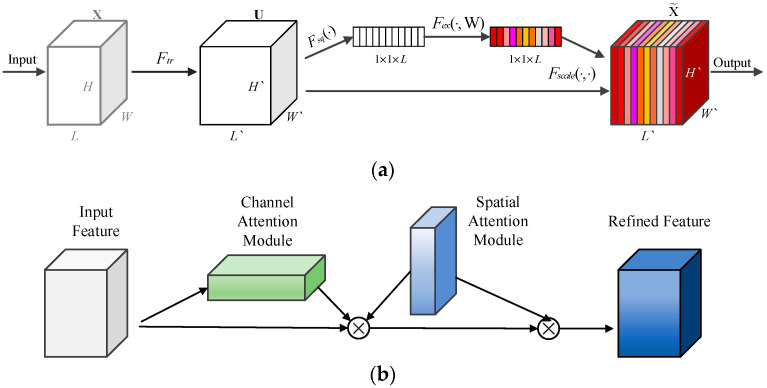
Schematic diagram of the principles of the two attentional mechanisms. (**a**) SE Net structure diagram. (This image was provided by Dr. Hu Jie of Momenta); (**b**) CBAM structure diagram. (This image was provided by Dr. Woo Sanghyun and Prof. Kweon In So of Korea Adv Inst Sci and Technol).

The SE Net attention mechanism [24,25], mainly by establishing the correlation between channels, automatically obtains the importance of each feature channel, according to which the useful features are promoted and irrelevant features are suppressed to achieve adaptive recalibration of channel orientation. Its structure is shown in Figure 5a. Initially, the feature map acquired by convolution is processed to obtain a one-dimensional vector with the same number of channels as the evaluation score of each channel, and then the modified scores are weighted to the corresponding channels by multiplication, and finally the output result is calculated.

CBAM attention mechanism [26], contains 2 independent submodules: the Channel Attention Module (CAM) and the Spatial Attention Module (SAM), both of which perform Attention on the channel and space, respectively, which can save parameters and computational power. The structure is shown in Figure 5b.

Both are easily integrated into existing network architectures as plug-and-play modules. In this paper, the SE Net and CBAM attention mechanisms are inserted in forward () for feature maps output by YOLOX_s backbone network, as shown in Figure 4.

#### 2.3.2. Confidence Loss Function Optimization

The loss function is used to evaluate the degree of difference between the predicted and true values of the model, and usually the smaller the loss function, the better the performance of the model. The original confidence prediction function is a binary Cross-entropy loss function, which is mostly applied to binary classification tasks with the following equation.

(1) $$Loss=-\frac{1}{N}\sum_{i=1}^{N}y_{i}\cdot \log[p(y_{i})]+(1-y_{i})\cdot \log[1-p(y_{i})]$$

In Equation (1), $y_{i}=\left\{0,1\right\}$ is the binary label and $y_{i}=\left\{0,1\right\}$ is the probability that the output belongs to the label $y_{i}$. When $p(y_{i})=1$, $p(y_{i})$ is expected to become large enough. When $y_{i}$ is a positive case, $p(y_{i})=1$, the loss is zero. When $p(y_{i})$ tends to zero, $\log p(y_{i})$ tends to negative infinity, the loss is extremely large. From the cross-entropy loss function, we know that for positive samples, the higher the cross-entropy output probability, the smaller the loss. For negative samples, the smaller the output probability is, the smaller the loss is. Since the loss function is very slow and may not even be optimized to the optimum during iterations with a large number of simple samples, the binary cross-entropy loss is considered to be replaced by Focalloss or Varifocalloss.

1. Focalloss function

The useless easily separable negative samples in the confidence prediction loss process cause the model to fail in learning and only discriminate the background without objects but not the specific objects. For the category imbalance problem, the Focalloss function proposed by Tsung et al. [27] makes the model more focused on such indistinguishable samples thus reducing the effect of simple samples. It is defined as follows.

(2) $$FL(p_{t})=-\alpha_{t}{\left(1-p_{t}\right)}^{\gamma }\log(p_{t})$$

In Equation (2), ${\left(1-p_{t}\right)}^{\gamma }$ is the adjustment factor, γ is the adjustable focusing parameter. When γ=0, Focalloss function becomes the cross-entropy loss function. When γ>0, the loss of easy classification samples is reduced and the model focuses more on difficult and misclassified samples. When γ increasing, the effect of the adjustment factor also increases.

2. Varifocalloss function

In past research work, the classification score is usually used or combined with the IoU-based localization score as the basis for ranking. This approach is unreliable and can even impair detection performance. Varifocalloss, proposed by Zhang Haoyang et al. [28], is mainly used to train dense object detectors to predict IACS. It is defined as follows.

(3) $$\begin{array}{l} VFL(p,q)=\\ \left\{\begin{array}{l} -q[q\log(p)+(1-q)log(1-q)]\\ -\alpha p^{\gamma }\log(1-p) \end{array}\right.\begin{array}{c} q>0\\ q=0 \end{array} \end{array}$$

In Equation (3), p is used to predict the IACS score, q stands for target IoU score. Set q to IoU between bbox and gtbox. For negative samples, the q value of the training target is 0 for all categories. Varifocalloss will predict the classification score and IACS score. $p^{\gamma }$ is used to effectively reduce the loss of negative sample weights and maintain the original weights of positive samples. q is used to increase the loss weights of positive samples, making the training focus on high quality samples.

### 2.4. Platform System Design

#### 2.4.1. Web Detection Platform

**Figure 6:**
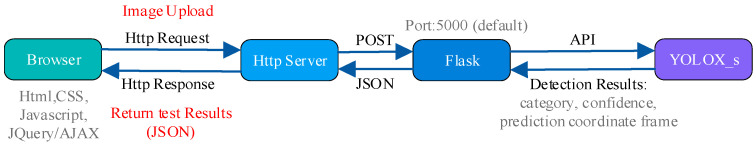
Flask-based Web-side YOLOX_s detection system framework.

Flask is a lightweight web application framework written in Python that is highly scalable and flexible, as shown in Figure 6. Jinja2 provides the template language and the Werkzeug module is responsible for configuring the web server gateway interface. Deploy improved Yolox_s grape pest detection module with Flask to achieve the function of user uploading local images for detection. The Web user service system is built by embedded database SQLite, including registration and login function, grape pest and disease encyclopedia model search, and pest and disease control suggestion function.

#### 2.4.2. Raspberry Pi Detection System Design

Raspberry Pi system includes wine grape pest and disease detection and remote interconnection to the Web [29,30,31].

**Figure 7:**
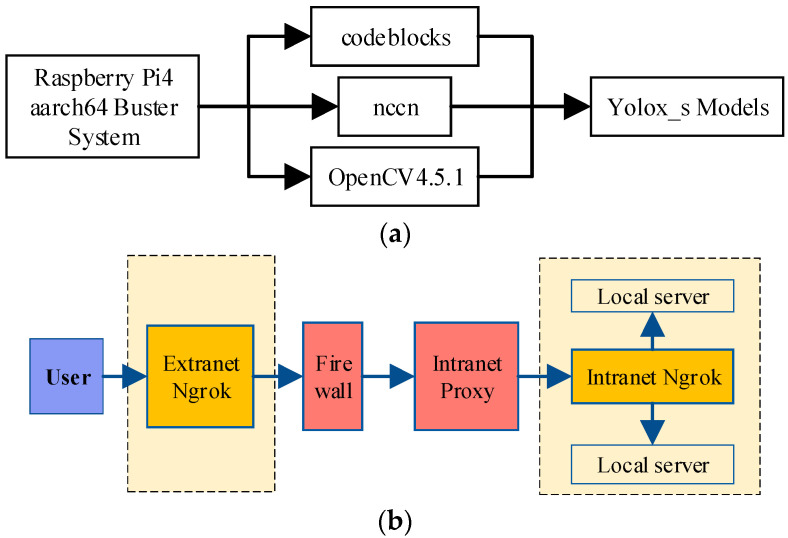
Platform Raspberry Pi platform build. (**a**) Implementation flow of embedding YOLOX_s in Raspberry Pi system. (**b**) Raspberry Pi and Web platform interconnection channel.

Under the Raspberry Pi4 aarch64 Buster system, OpenCV4.5.1, ncnn deep learning framework, Gstreamer multimedia framework [32] and V4L2 driver framework are used to build the identification system. CSI cameras are used to detect grape pests and diseases. The system structure is shown in Figure 7a.

The motion open-source camera monitoring software in Linux is used to achieve remote monitoring function in the intranet. Establishing a channel between the public endpoint and the Web server through ngrok to achieve intranet server management and extranet access functions, as shown in Figure 7b. Finally, in the Raspberry Pi system environment, noVNC and websockify are used to build VNC remote desktop, while designing html layout on the web side to achieve remote interconnection with Raspberry Pi. Based on the web platform, remote desktop function users can call the Raspberry Pi CSI camera to get real-time images of the grape farm.

## 3. Results

### 3.1. Evaluation Indicators

**Figure 8:**
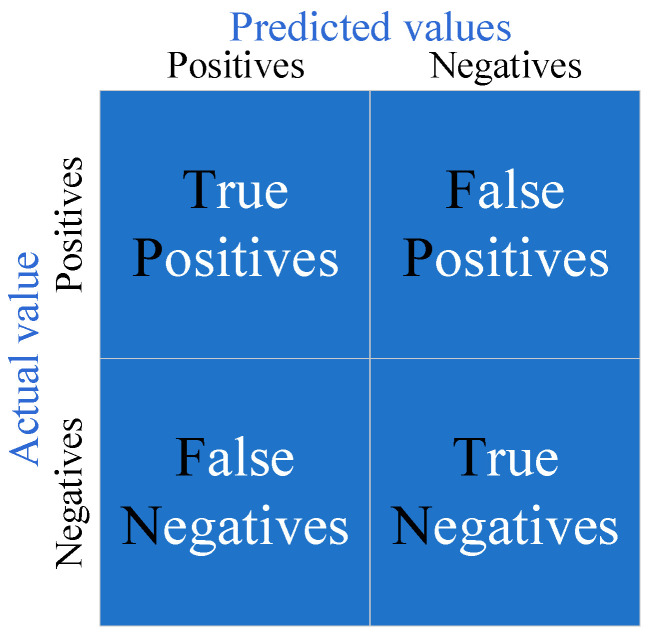
Principles of sample classification.

For the binary classification problem, the samples can be classified into four cases based on the combination of the sample true category and the learner prediction category: true case (TP), false positive case (FP), true negative case (TN), and false negative case (FN). The classification results are shown in Figure 8. The performance of the improved YOLOX_s is measured quantitatively using the average precision (AP) and the average recall (AR).

Precision represents the ratio of correct samples to all predicted subsamples. Recall is the proportion of samples that match the actual in the predicted correct sample to the total number of samples in the predicted correct sample. Precision (P) and recall (R) are defined as in Equations (4) and (5).

(4) $$Precision=\frac{TP}{TP+FP}$$

(5) $$Recall=\frac{TP}{TP+FN}$$

The performance of the model is determined by both precision and recall.

When the sample category is more homogeneous, the average precision AP is a comprehensive index to balance the two. With precision as the vertical axis and recall as the horizontal axis, a precision-recall curve (P-R Curve), can be obtained. The value of AP is the area of the closed graph formed by this curve and the horizontal axis in the interval of [0, 1].

(6) $$AP=\int_{0}^{1}P(R)dR$$

When there are many kinds of sample categories, mAP evaluates the performance of the model. The formula uses N to denote the kind of samples. Summing and then averaging the APs for each species, we can obtain the mean average precision (mAP), as in Equation (7).

(7) $$mAP=\frac{\sum_{i=1}^{N}AP_{i}}{N}$$

IoU is a criterion that defines the accuracy of target objective detection, which is calculated by the overlap ratio between the predicted bounding box and the true bounding box, as in Equation (8). The predicted results are considered valid when the value of IoU is greater than 0.5.

(8) $$IOU=\frac{Soverlap}{Sunion}$$

The average recall (AR) is defined as taking the maximum value of recall and then averaging it for different IoU of samples of the same category. The mean average recall (mAR) is defined as summing and then averaging the AR for each sample category under multiple samples.

The mAP when exceeding the IoU threshold of 0.5 is defined as the accuracy determination criterion. The mAR at the IOU confidence interval of [0.5, 0.95] was defined as the outcome quality determination criterion. The mAP priority reference level is defined to be higher than the mAR, and both are jointly used to measure our performance of improving YOLOX_s.

### 3.2. Comparison of Optimized Models

Eight optimization schemes were arranged and combined according to the optimization methods mentioned above, namely: inserting only SE module, inserting only CBAM module, replacing only Focalloss, replacing only Varifocalloss, SE + Focalloss, SE + Varifocalloss, CBAM + Focalloss, CBAM + Varifocalloss. The nine optimization models are trained in the training set of the custom dataset and tested one by one in the validation set to find the optimal solution, while the original YOLOX_s network is added for comparison. The experimental results are shown in Table 2.

The experimental results showed that compared with the original YOLOX_s, the average precision (mAP) increased by 2.5%, the mean average recall (mAR) increased by 3%, the time increment was 0.02 ms, and the detection speed did not decrease significantly after inserting CBAM attention mechanism. The recall rates of Focalloss, Varifocalloss, and CBAM + Varifocalloss are improved by 0.3%, 0.8% and 0.16%, respectively, but only the sample detection coverage is improved and the precision and speed are decreased. The other optimization combination methods failed to achieve good optimization results for each index. Therefore, this paper proposes to select CBAM + YOLOX_s as the final improved network and conduct training and validation.

### 3.3. Optimization Model Validation Based on the Overall Dataset

**Figure 9:**
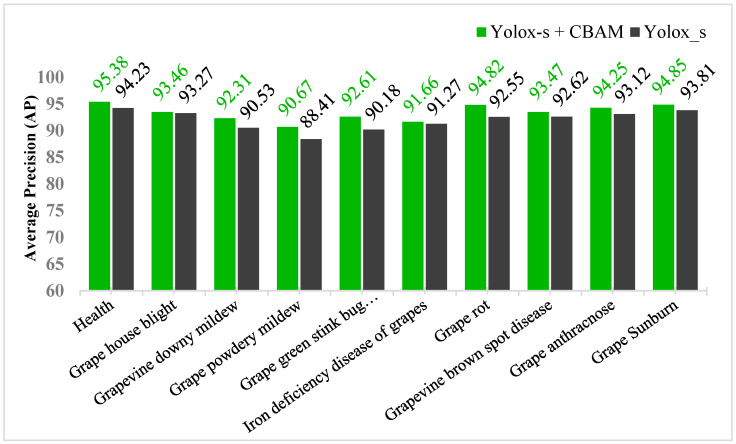
Comparison of the validation results of the optimization model on the overall dataset.

In order to make the improved YOLOX_s training model have better recognition performance, the training set of the overall dataset mentioned before is chosen to train the CBAM + YOLOX_s network, and finally the validation is performed in the validation set of the overall training set. The original YOLOX_s network was also added to the same training and configuration environment for control validation, and the experimental results are shown in Figure 9.

The test results showed that the mean average precision (mAP) of the original Yolox_s network was 92.00% and the mAP of Yolox_s + CBAM was 93.35%. Among all the items tested, grape sunburn had the highest detection accuracy of 94.85%. This is due to the fact that the disease is characterized by a brown color of the grape fruit and the differences are extremely pronounced leading to a high accuracy of identification. The worst detection was for grape powdery mildew, with an accuracy of 90.67%. This disease is characterized by a layer of white spots attached to the leaves, which is further weakened in sunlight conditions, resulting in poor identification accuracy. It is difficult to improve on the original detection accuracy of 92% in an unstructured natural environment. Taken as a whole, it is difficult to go up in unstructured natural environments based on the original detection accuracy of 92%. The overall recognition accuracy of this optimized model is improved by 1.35% over the original YOLO_s model, with significant optimization effect and better robustness in practical applications.

### 3.4. Platform Functionality Testing

#### 3.4.1. Web Platform Testing

**Figure 10:**
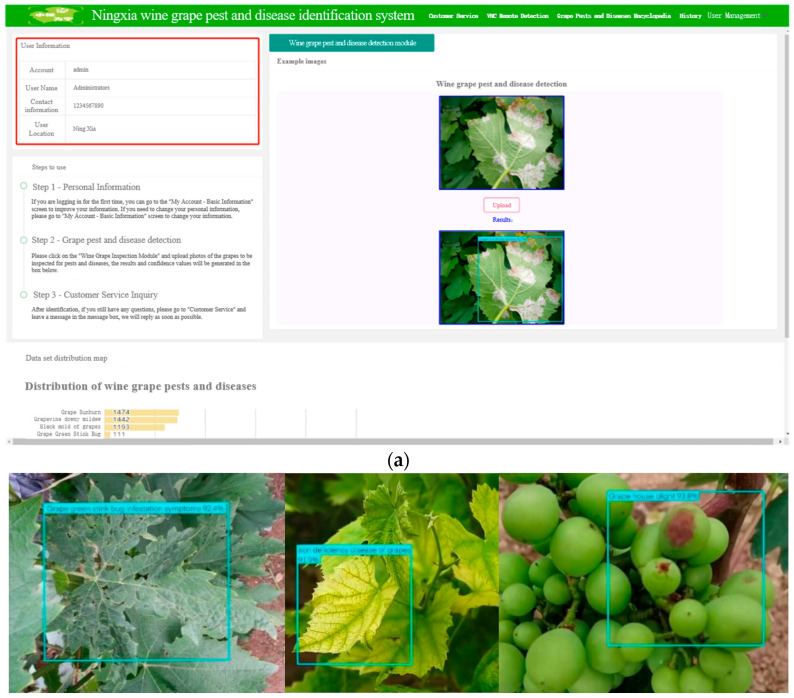

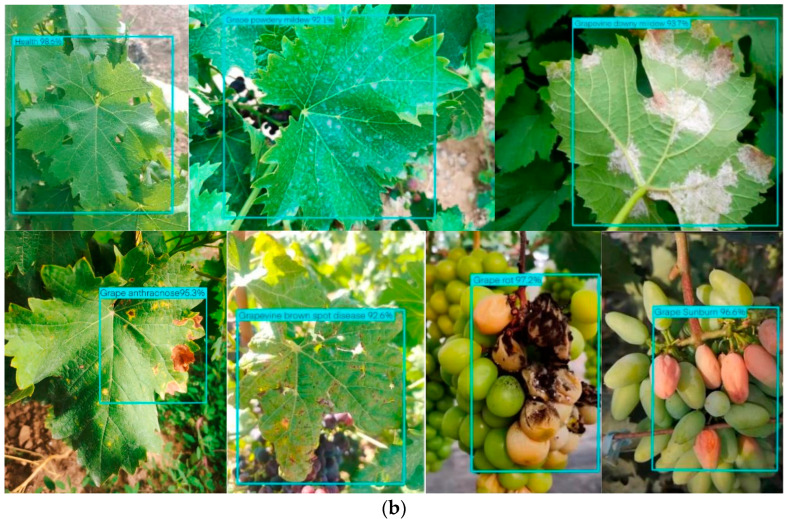
Functional testing of grape pest and disease detection on a web-based platform. (**a**) Web detection platform user interface. (**b**) Detection results of healthy leaf and 9 types of pest and disease grape leaves.

Uploading local pictures for pest and disease detection on the Web platform, the location of grape pests and diseases will be marked by the system with blue wireframes. The text is labeled with the type of pest and the number represents the probability that it may be that type of pest. The results of the platform test are shown in Figure 10a. The results of the detection of ten types of grape leaves are shown in Figure 10b.

#### 3.4.2. Raspberry Pi Field Testing

**Figure 11:**
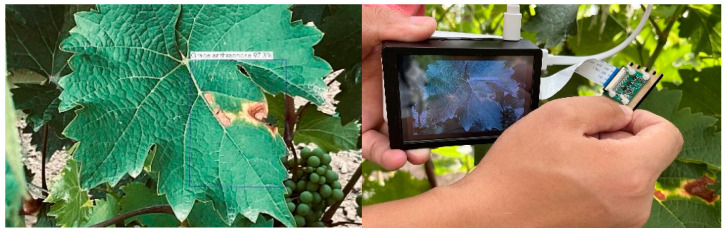
Verification of the Raspberry Pi field recognition function.

Pest and disease detection is performed in the field by hand-held raspberry parties equipped with CSI cameras on grape leaves, and disease information and probabilities are shown in the on-screen display. The detection effect is shown in Figure 11. The experimental results show that the function works properly and can meet the needs of field detection while having portability.

#### 3.4.3. Remote Interconnection Detection

**Figure 12:**
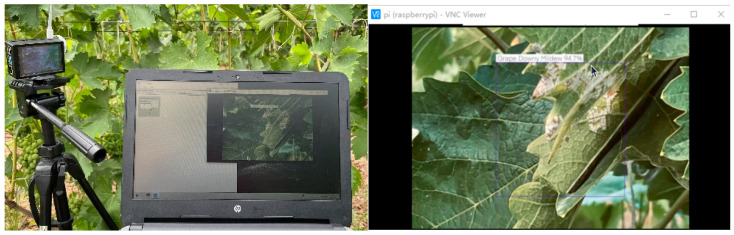
Functional validation of remote real-time detection of grape pests and diseases.

The location of the Raspberry Pi is arranged in the field and ensure that the Raspberry Pi and the computer are in the same LAN environment. Users can monitor and control the Raspberry Pi in real time by enabling the VNC program on the computer. The Raspberry Pi will transmit the images taken by the CSI camera to the remote user’s computer desktop and mark the location and information of the pests and diseases present in the images. The effect of the remote display is shown in Figure 12.

The results show that both platforms can effectively detect pests and diseases of Ningxia wine grapes under natural environment and can meet the needs of three kinds of detection scenarios: on-site detection, Web-based detection, and remote real-time detection.

## 4. Conclusions and Discussion

In this paper, we designed a pest and disease detection platform for wine grapes in the Ningxia region, which can effectively detect 1 pest and 8 diseases. The public dataset, the custom dataset, and the overall dataset are constructed separately, and the appropriate dataset is selected from them according to the characteristics of different datasets to meet the needs of the experimental process.

For the target detection network, the recognized accuracy of both MobileNet V2 and YOLOX_s are compared under the training of public dataset to select the best base network. By exploring the effects of adding SE and CBAM attention mechanism modules and replacing FocalLoss and Varifocalloss loss functions on the optimization results, the best recognition network, YOLOX_s + CBAM, is identified from nine schemes. After comparing with the original YOLOX_s network, it can be seen that the optimized network has a significant improvement in the mean average accuracy.

To build the platform, Yolox_s model is deployed within the Flask framework to achieve the web-side pest identification function. The platform service module is built based on SQLite, an embedded database, to realize user login and pest and disease encyclopedia query. A Yolox_s model to ncnn deep learning framework in mobile side is added to realize the recognition of pests and diseases in Raspberry Pi system environment. Based on noVNC, websockify achieves Raspberry Pi VNC remote desktop remote desktop, through ngrok to establish a public endpoint and the local running Web server channel to achieve the interaction between the Web and Raspberry Pi.

Users can upload local pictures for inspection through the Web platform, or hold a Raspberry Pi equipped with a CSI camera to inspect the grapes in the field on site, or they can arrange the Raspberry Pi to the field for remote interconnection and inspection on the PC side. The platform is designed to meet the detection needs of different scenarios. This study greatly improves the efficiency of pest detection, improves grape yield and quality, reduces labor intensity, saves labor costs, solves the technical problems of intelligent pest control in the wine grape industry in Ningxia region, and plays a positive role in promoting the realization of agricultural intelligence and automation in the future.

## Figures and Tables

**Table 1:** Three types of dataset characteristics.

Data Set Type	Types of Pests and Diseases (Health /Disease/Insect)	Number of Samples (Health/Disease/Insect)
Public Dataset	13 (1/11/1)	42150 (3570/35620/2960)
Custom Dataset	10 (1/8/1)	21850 (2450/17470/1930)
Overall Dataset	10 (1/8/1)	32760 (6020/24810/1930)

**Table 2:** Comparison of average precision, average recall and average NMS for 8 network optimization results.

No.	CombinationType	Optimize Portfolio Content	Mean Average Precision (mAP) @ [IoU = 0.50|area = all|maxDets = 100]	Mean Average Recall (mAR) @ [IoU = 0.50:0.95 |area = large|max Dets = 100]	Average NMS Time (ms)
1	Initial	YOLOX_s	0.919	0.945	1.41
2	Add attention mechanism	Insert SE Net	0.862 (−0.057)	0.921 (−0.024)	1.70 (+0.29)
3		Insert CBAM	0.944 (+0.025)	0.948 (+0.003)	1.43 (+0.02)
4	Confidence prediction loss replacement	Binary Cross-entropy loss replacement with Focalloss	0.913 (−0.006)	0.953 (+0.008)	2.98 (+1.57)
5		Binary Cross-entropy loss replacement by Varifocalloss	0.915 (−0.004)	0.961 (+0.016)	2.26 (+0.85)
6	Insert attention mechanism + confidence prediction loss function replacement	SE + Focalloss	0.872 (−0.047)	0.900 (−0.045)	3.01 (+1.60)
7		SE + Varifocalloss	0.890 (−0.029)	0.893 (−0.052)	2.23 (+0.82)
8		CBAM + Focalloss	0.901 (−0.018)	0.935 (−0.010)	2.99 (+1.58)
9		CBAM + Varifocalloss	0.896 (−0.023)	0.958 (+0.013)	2.28 (+0.87)

## Data Availability

Data and code are available upon request.
